# *Pseudomona*s Endocarditis with an unstable phenotype: the challenges of isolate characterization and Carbapenem stewardship with a partial review of the literature

**DOI:** 10.1186/s13756-017-0245-5

**Published:** 2017-08-29

**Authors:** Emil Lesho, Erik Snesrud, Yoon Kwak, Ana Ong, Rosslyn Maybank, Maryrose Laguio-Vila, Ann R. Falsey, Mary Hinkle

**Affiliations:** 1 Infectious Diseases Unit, Rochester Regional Health, Rochester, NY USA; 2 Multidrug-resistant Organism Repository and Surveillance Network, Walter Reed Army Institute of Research, Silver Spring, MD USA; 3 Department of Medicine, University of Rochester School of Medicine, Rochester, NY USA

**Keywords:** *Pseudomonas aeruginosa*, Endocarditis, Stewardship, Whole-genome sequencing

## Abstract

**Background:**

*Pseudomonas* endocarditis is exceedingly rare, especially in patients without predisposing risks. We present such a case that included unexpected switches in antibacterial resistance profiles in two *Pseudomonas aeruginosa* (PA) strains with the same whole-genome sequence. The case also involved diagnostic and treatment challenges, such as issues with automated testing platforms, choosing the optimal aminoglycoside, minimizing unnecessary carbapenem exposure, and the need for faster, more informative laboratory tests.

**Case presentation:**

On hospital day one (HD-1) a cefepime and piperacillin-tazobactam (FEP-TZP)-susceptible *P. aeruginosa* was isolated from the bloodstream of a 62-year-old man admitted for evaluation of possible endocarditis and treated with gentamicin and cefepime. On HD-2, his antibiotic regimen was changed to tobramycin and cefepime. On HD-11, he underwent aortic valve replacement, and *P. aeruginosa* was isolated from the explanted valve. Unexpectedly, it was FEP-TZP-resistant, so cefepime was switched to meropenem. On HD-14, in preparation for whole-genome sequencing (WGS), valve and blood isolates were removed from cryo-storage, re-cultured, and simultaneously tested with the same platforms, reagents, and inoculations previously used. Curiously, the valve isolate was now FEP-TZP-susceptible. WGS revealed that both isolates were phylogenetically identical, differing by a single nucleotide in a chemotaxis-encoding gene. They also contained the same resistance genes (*bla*_ADC35_, *aph(3′)-II*, *bla*_OXA-50_, *catB7*, *fosA).*

**Conclusion:**

Repeated testing on alternate platforms and WGS did not definitively determine the resistance mechanism(s), which in this case, is most likely unstable de-repression of a chromosomal AmpC β-lactamase, porin alterations, or efflux upregulation, with reversion to baseline (non-efflux) transcription. Although sub-culture on specialized media to select for less fit (more resistant) colonies, followed by transcriptome analysis, and multiple sequence alignment, might have revealed the mechanism and better informed the optimal choice of β-lactam, such approaches are neither rapid, nor feasible for hospital laboratories. In this era of escalating drug resistance and dwindling antibiotics, use of the most potent anti-pseudomonals must be balanced with stewardship. Clinicians need access to validated genomic correlates of resistance, and faster, more informative diagnostics. Therefore, we placed these isolates and their sequences in the public domain for inclusion in the *Pseudomonas* pan-genome and database projects for further countermeasure development.

## Background

*Pseudomona*s endocarditis is exceedingly rare and potentially devastating [1, 2]. Neurological sequelae, annular and splenic abscesses, and congestive heart failure frequently complicate *Pseudomonas* endocarditis [1]. All non-HACEK Gram-negative aerobic bacteria (species other than *Haemophilus* species, *Actinobacillus actinomycetemcomitans, Cardiobacterium hominis, Eikenella corrodens,* or *Kingella* species) combined accounted for only 1.8% of all causes of endocarditis in a multinational database of over 2700 patients in 18 countries. *Pseudomonas* comprised only a small fraction of that 1.8% [1]. In one of the largest U.S. reports, most cases were associated with intravenous drug use, implanted prosthetic cardiac valves or endovascular devices [2]. Early surgery is usually indicated in patients with left sided native valve endocarditis with one or more of the following: persistently positive blood cultures; valve dysfunction; paravalvular extension; annular abscess; destructive or penetrating lesions; heart block; infections with difficult to treat or highly resistant pathogens and recurrence of bacteremia recurs after completion of the initial therapy [2, 3].

Whole-genome sequencing is commonly applied to analyze and understand the genotype and phenotype of an organism, and at least 27 complete genome sequences of *P. aeruginosa* are now available in GenBank [4–6]. Researchers established a genomic pool of sequenced isolates referred to as the pan-genome of *P. aeruginosa* [4, 6, 7]. Currently it consists of more than 4000 core genes and 10,000 accessory genes, along with 30,000 rare genes that are found in only a few strains or clonal complexes [4, 7]. Most of the reports of genomically characterized *Pseudomonas* isolates are either laboratory adapted strains, or strains form the respiratory tracts of patients with cystic fibrosis. Against this backdrop, there are calls for more genomes, especially those from severe *P. aeruginosa infections* in non-immunocompromised patients and patients without cystic fibrosis. [4, 7]. There are also ongoing efforts to establish a public database analogous to a GenBank solely dedicated to antimicrobial resistant pathogens, to aid in the development of diagnostic and therapeutic countermeasures [7, 8].

We could find no reports leveraging whole-genome sequencing (WGS) to characterize an apparently unstable *Pseudomonas aeruginosa* phenotype in a patient with *Pseudomonas* endocarditis and no predisposing risk factors. To highlight diagnostic and treatment challenges such as choice of the optimal aminoglycoside, minimizing unnecessary carbapenem exposure, issues with automated testing platforms, and the need for more rapid and informative laboratory tests, we present such a case involving two unexpected events that occurred early in the course of treatment.

## Case presentation

A 62-year-old Caucasian man with a history of lung cancer and recurrent pyelonephritis was admitted for work up for possible endocarditis because of fevers and *Pseudomonas* bacteremia. He had none of the previously mentioned risk factors for *Pseudomonas* endocarditis. One month prior to admission, he was treated in another state for pyelonephritis and prostatitis with piperacillin-tazobactam (1–3 days), followed by an unknown fluoroquinolone (approximately 10–14 days). Urine culture results from those events were not available. On admission, his serum chemistries, liver associated enzymes, and creatinine were normal. His complete blood count was notable for a hemoglobin of 11 g/dl (13–18 g/dl), and a hematocrit of 37% (40–52%). Computerized tomography of the chest abdomen and pelvis revealed diffuse emphysematous changes, small bilateral pleural effusions, minimal abdominal ascites, and a wedge-shaped zone of diminished attenuation in the right kidney. Treatment with gentamicin and cefepime was initiated. The *P. aeruginosa* isolated from his bloodstream was susceptible to ceftazidime (minimum inhibitory concentration (MIC) =4 μg/ml), cefepime (2 μg/ml), ciprofloxacin (<0.25 μg/ml), piperacillin-tazobactam (8 μg/ml; zone diameter > 21 mm), ticarcillin-clavulanate (32 μg/ml), gentamicin (2 μg/ml), tobramycin (<1 μg/ml), amikacin (</=2 μg/ml), imipenem (2 μg/ml), and meropenem (0.5 μg/ml). On HD-2, infectious diseases consultation was obtained, and therapy was changed to 2 g cefepime every 8 h, plus 7 mg/kg of tobramycin daily. Subsequently, blood cultures became negative and transesophageal echocardiogram revealed a mobile mass on the aortic valve and severe aortic regurgitation. On HD-11, the patient underwent aortic valve replacement, and *P. aeruginosa* was isolated from the excised valve tissue. Unexpectedly, the valve isolate was resistant to ceftazidime (>/=64 μg/ml), cefepime (MIC 32 μg/ml), piperacillin-tazobactam (128 μg/ml, zone diameter 21 mm); and ticarcillin-clavulanate (128 μg/ml). MICs to all the other previously mentioned antibiotics were the same as the MICs of bloodstream isolate. Both the valve and blood isolates were tested on the same platform (Vitek ®2, bioMérieux, France), with the same card-AST GN69 (bioMérieux, France) from the same lot (5,890,118,103), using the same inoculation protocols. Furthermore, all results for piperacillin–tazobactam are confirmed with supplemental disk diffusion (Kirby–Bauer) testing before being released. The zone diameter of piperacillin-tazobactam on the blood isolate was >21 mm (sensitive) and 12 mm (resistant) on the valve isolate, which were concordant with the Vitek 2 results. We debated whether the bloodstream or the excised valve isolate should dictate the choice of β-lactam, replaced the cefepime with meropenem, and continued the tobramycin.

To assess genetic relatedness and resistance gene content, both isolates underwent whole-genome sequencing (WGS) on a MiSeq bench-top sequencer (Illumina, San Diego, CA) using methods described previously and ResFinder [9, 10]. On HD14 In preparation for sequencing, both isolates were thawed, re-cultured on the same media (MacConkey agar (Millipore-Sigma, Temecula, CA)), and simultaneously tested on the same platform using the same card from the same lot as was used in the previous tests. Unexpectedly, the cefepime MIC of the valve isolate had decreased from 32 μg/ml–4 μg/ml, and the piperacillin-tazobactam MICs had fallen from >128 to 8 μg/ml. In the interest of antimicrobial stewardship, and cognizant of the fact that in one series more than 50% of the patients who started with a fully carbapenem susceptible isolate had a >/=4-fold increase in carbapenem MIC during carbapenem therapy [11], we again debated the optimum β-lactam and whether cefepime should replace meropenem for the remaining 3.5 weeks of therapy.

**Fig. 1:**
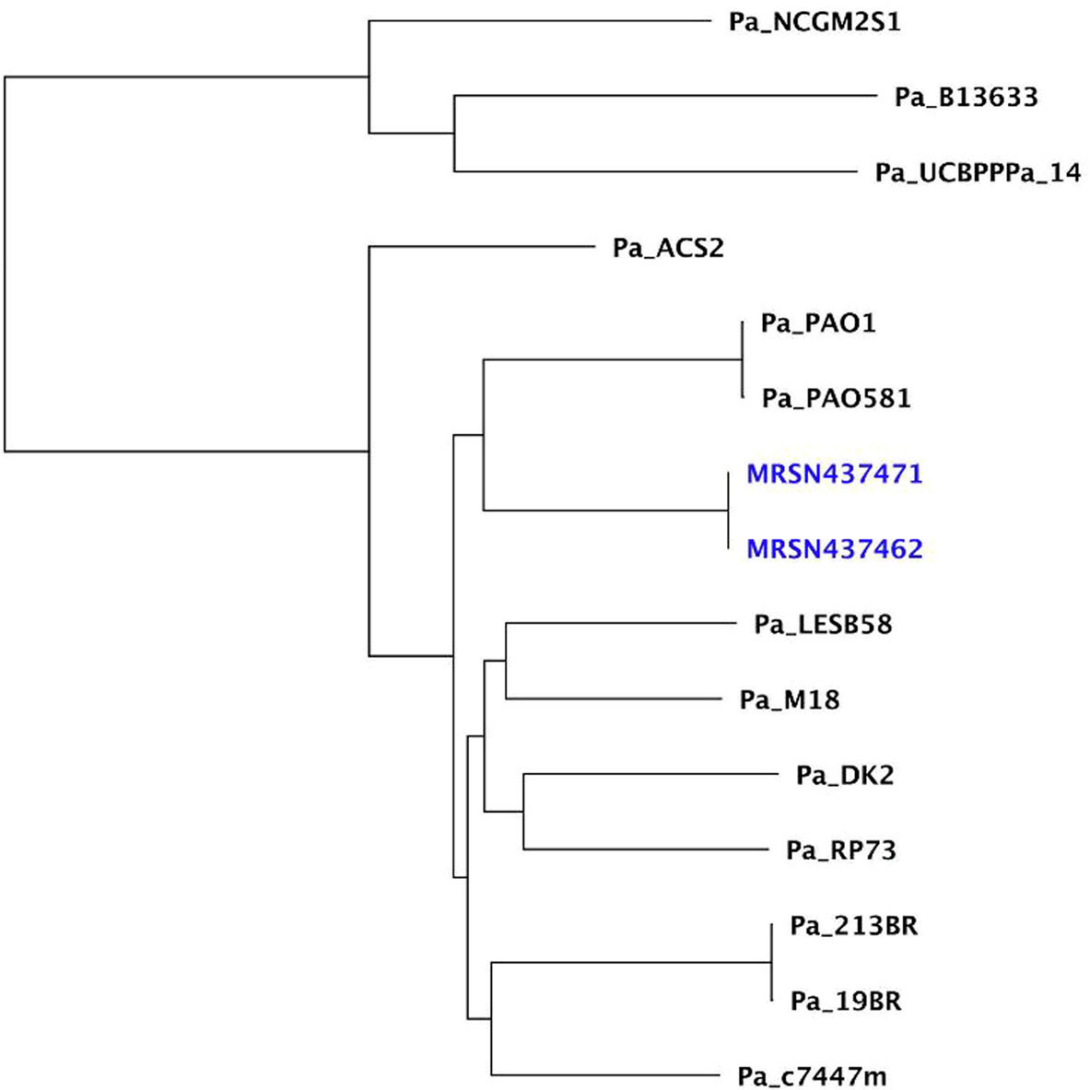
Core genome phylogeny of *Pseudomonas* isolates. Dendrogram generated from the core genome sequence of the *P. aeruginosa* isolates from this patient (Blue text) and other *P. aeruginosa* sequences from Genbank (Black text). Branch lengths are indicative of strain relatedness. MRSN 437462 = valve isolate. MRSN 437471 = blood isolate

WGS revealed that the valve and bloodstream isolates were identical –differing by only one nucleotide in the cheA gene, resulting in truncation and inactivation of CheA which is involved with motility and chemotaxis [12, 13]. Both isolates belonged to multi-locus sequence type (ST) 162 and contained the same set of resistance genes (*bla*_ADC35_ *, aph(3′)-II, blaOXA*_*−50*_ *, catB7,* and *fosA*)*.* There are very few reports of *P. aeruginosa* ST-162. Of those reports available, ST162 has been associated with *Klebsiella pneumonia* carbapenemase producing strains in Argentina, and a VIM-2 producing strain in Algeria [14, 15] At the whole-genome level, the isolates in our patient were most closely related to reference strains PA_O1 and PA_O581, followed by strain PA_LESB58 (Fig. 1). PA_O1 is a spontaneous chloramphenicol-resistant mutant derived from of the original strain that had been isolated in 1954 from a wound in Melbourne, Australia [16, 17]. PA_O581 is an alginate producing mucoid strain isolated from the sputa of cystic fibrosis patients in Scotland [18, 19]. PA_LESB58 is the hyper-virulent ‘Liverpool Epidemic Strain’ from an outbreak in pediatric cystic fibrosis clinic in England. PA_LESB58 is a strain that overproduces pyocyanin and other quorum sensing exoproducts and has been associated with transmissibility, dominance over other *P. aeruginosa* populations in CF airways, increased morbidity and mortality, and causing infections in healthy parents (and a pet cat) of children with cystic fibrosis [19–24]. In light of this potential for increased virulence, and the uncertainty of the resistance profiles, meropenem and tobramycin were continued. Two months after completing 6 weeks of total antibiotic therapy, the patient had no evidence of endocarditis and was doing well.

## Discussion

There are no prospective trial data that define the optimal antimicrobial management for the treatment of endocarditis caused by non-HACEK Gram-negative aerobic bacilli [3]. Furthermore, little is known about how to optimize chemotherapy for serious *P. aeruginosa* infections [25]. The goals of antibiotic therapy are to rapidly kill the bacteria to facilitate granulocyte function, and to suppress the amplification of resistant sub-populations. Single and/or multi-agent dosing regimens, guided by pharmacokinetic-pharmacodynamic targets and mathematical modeling, help achieve those goals [25, 26]. Such regimens include a meropenem –levofloxacin combination for *Pseudomonas* pneumonia [27], 2 g cefepime every 8 h, plus 7 mg/kg of tobramycin daily for *Pseudomonas* endocarditis [2, 25], and possibly imipenem or doripenem with ciprofloxacin [26] . Although gentamicin is recommended for certain types of endocarditis caused by *Staphylococcus aureus* and *Enterococcus* [3], clinicians may not recall that tobramycin (not gentamicin) is specifically recommended for pseudomonal endocarditis [2, 3].

In addition to illustrating treatment challenges, this case highlights the need for more informative laboratory tests with clinically relevant turnaround times. Even with WGS and repeated testing on equivalent and supplemental platforms we could not definitively determine the resistance mechanism(s), or which phenotype should dictate the choice of a carbapenem versus a non-carbapenem.

With more data and experience, one possible carbapenem-sparing regimen in the future might include either of the two newest agents with activity against multidrug-resistant *Pseudomonas*. These are ceftolazane- tazobactam and ceftaroline-avibactam. Avibactam is a synthetic non-β-lactam β-lactamase inhibitor that inhibits the Ambler class A and C β-lactamases and some Ambler class D including *Klebsiella pneumoniae* carbapenemases, AmpC, and OXA-48-like carbapenemases. It is important to remember avibactam does not inhibit metallo-β-lactamases such as VIM and NDM variants [28]. Ceftolazane-tazobactam retains in vitro activity against *Pseudomonas* strains with the three most common resistance mechanisms discussed below [29] and was the most potent antispsueudomonal in two studies [30, 31].

The ability of *Pseudomonas* to rapidly develop resistance to multiple classes of antibiotics during the course of treating a patient represents one of the most difficult challenges infectious disease clinicians face [26]. Perhaps no genera of bacteria has such a wide array of both extrinsic (imported) and intrinsic chromosomal mechanisms at its disposal. Lister, et al. provide a comprehensive review of AmpC, OprD, and efflux-mediated resistance mechanisms, including the coregulation of multiple mechanisms and strategies for preventing the emergence of resistant sub-populations [26]. Resistance to extended spectrum cephalosporins in *Pseudomonas aeruginosa* most often occurs by de- repression of the chromosomal AmpC β-lactamase, porin alterations resulting in impermeability of the outer membrane, and increased efflux [32, 33]. Occasionally resistance in *Pseudomonas* is due to acquisition of oxacillinase encoding genes such as *bla*_OXA-35_, *bla*_OXA1_, *bla*_OXA4_ [34]. Isolates with those genes usually display a phenotype that is resistant to cefepime but susceptible to ceftazidime [34]. Another mechanism that produces such a FEPs/CAZr phenotype is overexpression of mexY which codes for the multi-drug efflux system MexXY-OprM [35]. Some double efflux mutants (both mexAB-oprM and mexXY) have been described that are resistant to cefepime, ticarcillin, and aztreonam [35]. Aminoglycosides alone or in combination with fluoroquinolones, can select for MexXY gain of efflux mutants in vitro [35].

Since the isolates in this case were genetically identical, we inferred that the changing phenotype was not due to acquisition and subsequent loss of oxacillinase encoding genes such as *bla*_OXA-35_, *bla*_OXA1_, or *bla*_OXA4_. Furthermore, isolates with those genes usually are resistant to cefepime but susceptible to ceftazidime [34, 35], and the valve isolate in our patient was ceftazidime resistant.

We surmised that resistance was either due to unstable de-repression of a chromosomal AmpC enzyme, porin alterations resulting in membrane impermeability, or increased efflux.

The isolates in the freezer were not under selection pressure from ongoing cefepime exposure and might have reverted to baseline (non-efflux) transcription, with the less-fit resistant phenotype being outcompeted by the susceptible population. In retrospect, perhaps we should have used selective cefepime containing media during sub-culturing from the freezer, and that is another potential teaching point. However, we wanted to replicate the original testing conditions, and believed using specialized media would create selection bias. It is interesting to speculate that the valve isolate might have evolved into a more non-planktonic or sessile form by shedding or inactivating its chemotactic and motility machinery to limit any fitness cost and/or divert more of its resources to biofilm formation.

Although isolates in this case were not carbapenem resistant, such resistance in *Pseudomonas* is occurring more frequently [26, 36]. Carbapenemase encoding genes such as *bla*SPM, *bla*VIM, *bla*IMP and *bla*GIM on mobile genetic elements, are one of the most frequent mechanisms for such resistance [26, 36]. In contrast, published data have suggested that overproduction of AmpC does not play a discernible role in the development of carbapenem resistance among PA isolates [26]. Similarly, unlike their effect in *Enterobacteriaceae*, ESBLs alone do not result in a carbapenem resistant phenotype in *Pseudomonas* [26].

The Vitek 2 platform has been reported to produce variable results when testing *Pseudomonas* with cefepime and piperacillin-tazobactam [37], but we do not think this was the reason for the changing phenotype for several reasons. First, the differences we observed were on the order of many doubling dilutions. The Vitek results were confirmed and concordant with disk diffusion testing. Third, a more recent analysis of Vitek 2 performance using the newer (AST-GN69) cards (the same ones our hospital uses) wherein the TZP has been reformulated, were concordant with reference methods [38]. Additionally, an increase in the MICs of cefepime, piperacillin-tazobactam, and ticarcillin-clavulanate was biologically plausible given the antibiotics the patient previously received for the urinary tract infection. As the Vitek 2 is one of the three most widely used commercial automated platforms, others might face this or a similar question. Finally, some investigators have promoted replacing the MIC with the mutant prevention concentration (MPC), but the MPC not been adopted by clinical laboratories [26, 39–41]. A refinement of the MCP, the time in the MPC window, has been proposed. This is the time the serum drug concentration is above the concentration of drug that exerts minimal selection pressure and below the MPC [39].

Although WGS is increasingly acclaimed to have the potential to predict drug resistance, obviate the need for susceptibility breakpoints, and revolutionize the clinical practice of infectious disease [42–44], several barriers remain. These include lack of standardized quality control metrics (necessary for accrediting and comparing results), limitations of short-read sequencing (unable to close plasmids or other mobile genetic elements), insufficient bandwidth for sending sequence data (sequence data files are huge), and the limited availability of ultra-long-read platforms [42, 45]. Furthermore, for predicting resistance and susceptibility the current evidence base has recently been described as either “poor or non-existent” [8]. Nonetheless, WGS did provide insight into the possible mechanisms of resistance and the phylogenetic relatedness to the hyper virulent PA_LESB58 strain. This knowledge tipped the scales in favor or continuing the carbapenem instead of switching back to cefepime. Additionally, knowing that the strain was somewhat related to the transmissible PA_LESB58 epidemic strain, we alerted clinicians and infection control personnel to be vigilant for subsequent *Pseudomonas* infections in involved health care workers and nearby patients.

## Conclusion

*Pseudomonas* endocarditis is extremely rare, especially in patients such as the one here with no predisposing risk factors. This case, involving two unexpected events that occurred early in the course of treatment, highlights diagnostic and treatment challenges. These include issues with automated testing platforms, the need for more informative laboratory tests, choice of aminoglycoside, and minimizing unnecessary carbapenem exposure. Furthermore, pharmacologic optimization based on the susceptibility of the original clinical isolate does not always address the resistance emerging during therapy [26]. The reason for the changing phenotype could not be conclusively determined, even with extensive additional testing. It was not likely due to acquisition and subsequent loss of oxacillinase encoding genes. Instead it was probably due to unstable de-repression of a chromosomal AmpC enzyme, porin alteration or increased efflux with reversion to baseline (non-efflux) transcription in the absence of cefepime pressure during cryopreservation and in the standard McConkey culture media.

Sub-culture on specialized media to select for less fit (more resistant) colonies, followed by differential gene expression analysis and multiple sequence alignment, to look for specific porin mutations, might have revealed the mechanism and better informed the optimal choice of β-lactam. However, such approaches are neither rapid, nor feasible for most hospital laboratories. In this era of escalating drug resistance and dwindling antibiotics, use of the most potent anti-pseudomonals must be balanced with safe and appropriate stewardship. Such a balancing act requires clinicians have access to more informative and faster diagnostics. Given the dwindling number of antibiotics and escalating frequency of resistant strains, antibiotic alternatives or adjuncts are urgently needed. With more data from controlled trials, phage therapy might be one such adjunct. In an in vitro and an animal model single-dose phage therapy was active against *P. aeruginosa* endocarditis and synergistic when combined with cirpofloxacin [46, 47]. To support the development of diagnostic and therapeutic countermeasures, and contribute to the *Pseudomonas* pan-genome and *Pseudomonas* genome database [4, 6, 8], we have placed these isolates and their sequences in the public domain.

## Abbreviations

FEP: Cefepime
HD: Hospital day
MIC: Minimum inhibitory concentration
MPC: Mutant prevention concentration
non-HACEK: Species other than *Haemophilus* species, *Actinobacillus actinomycetemcomitans, Cardiobacterium hominis, Eikenella corrodens,* or *Kingella* species
PE: *Pseudomonas* endocarditis
TZP: Piperacillin-tazobactam
WGS: Whole-genome sequencing
